# Evaluation of serological assays for the diagnosis of HIV infection in adults

**DOI:** 10.4102/safp.v63i1.5316

**Published:** 2021-10-25

**Authors:** Avania Bangalee, Sachin Bhoora, Rivak Punchoo

**Affiliations:** 1 Department of Medical Virology, Faculty of Health Sciences, University of Pretoria, Pretoria, South Africa; 2 Tshwane Academic Division, National Health Laboratory Services, Tshwane, South Africa; 3 Department of Chemical Pathology, Faculty of Health Sciences, University of Pretoria, Pretoria, South Africa

**Keywords:** human immunodeficiency virus, HIV, enzyme immunoassay, enzyme-linked immunosorbent assay, EIA, ELISA, analytical error, HIV biomarkers, HIV diagnosis

## Abstract

Serological tests based on the enzyme immunoassay (EIA) are the primary tool for the diagnosis of human immunodeficiency virus (HIV) in adults and have rapidly evolved to quicker, affordable and more accurate test formats to detect early HIV infection. Second- and third-generation HIV rapid tests detect the immunoglobulin G (IgG) and immunoglobulin M (IgM) antibodies to the HIV and are used at the point of care and in HIV self-testing. The tests are affordable and accessible in state and private diagnostic laboratories. The present-day fourth- and fifth-generation EIAs can detect both p24 antigen and IgG and IgM HIV antibodies and thereby diagnose early HIV infection at approximately 2 weeks. The fourth- and fifth-generation EIAs also report sensitivity and specificity of more than 99%. The correct interpretation of HIV diagnosis of false-positive and false-negative EIA test results requires collaborative scrutiny of patient factors and laboratory test methodologies.

## Introduction

South Africa (SA) has one of the highest burdens of human immunodeficiency virus (HIV) infection in the world, and to achieve the 90-90-90 goals advocated by Joint United Nations Programme on HIV/AIDS (UNAIDS) accurate, early testing and linkage to care are required.^1,2^ Serological tests based on the enzyme immunoassay (EIA) have historically been the primary method for HIV diagnosis and currently remain the gold standard tool for diagnosing HIV infection in adults and children (older than 18 months).^3^ The evolution of HIV assay technology has resulted in quicker, affordable and improved test accuracy. Despite scientific advancement in HIV assay technology, clinicians are still challenged in a minority of patient cases by false-positive and false-negative HIV results.

## HIV biomarkers reflect evolving HIV infection

During the early phase of HIV infection after breach of the mucosal barrier, HIV infects target cells, for example, cluster of differentiation 4 (CD4) T lymphocytes. The primary viral amplification occurs in regional lymph nodes. This period is known as the eclipse phase (before the virus or antibodies are detected) and lasts approximately 10 days post-infection. Infected white blood cells and HIV virions then travel via the bloodstream to organs of the reticuloendothelial system and to lymphoid tissue in the gastrointestinal tract where secondary amplification occurs. This rapid replication and seeding of the virus results in very high ribonucleic acid (RNA) levels in blood, which becomes detectable from about 2 weeks post-infection.^4^ Between days 11 and 13 post-infection, the protein (p24) antigen (part of the core protein of HIV) may be detected in newly infected individuals. Thereafter, an immune response is mounted. The appearance of immunoglobulin M (IgM) after approximately 2 weeks is followed closely by immunoglobulin G (IgG) at about 3 to 4 weeks post-infection. The viral RNA then decreases to a ‘set point’ level.^5,6^ This marks the end of early HIV infection. Detection of HIV during this early period is important because of the clinical benefit of instituting early antiretroviral therapy.^7,8^

HIV is challenging to diagnose in the early ‘window period’ of infection. The window period is characterised by a lack of antibodies and p24 antigen.^9^ The median window period lasts approximately 18 days from infection and generally ranges between 10 and 24 days. Once antibody has appeared (seroconversion), antibody levels progressively increase over the next months and peak at 5–6 months. Thereafter, the antibody levels plateaux and remain fairly constant. Untreated, the infection evolves into advanced disease (Acquired Immunodeficiency Syndrome [AIDS]) characterised by opportunistic infections, a rise in HIV RNA levels and a gradual decline in HIV antibodies.^10^

## The evolution of EIA testing for HIV has narrowed the window period for screening and diagnosing HIV infection

**FIGURE 1:**
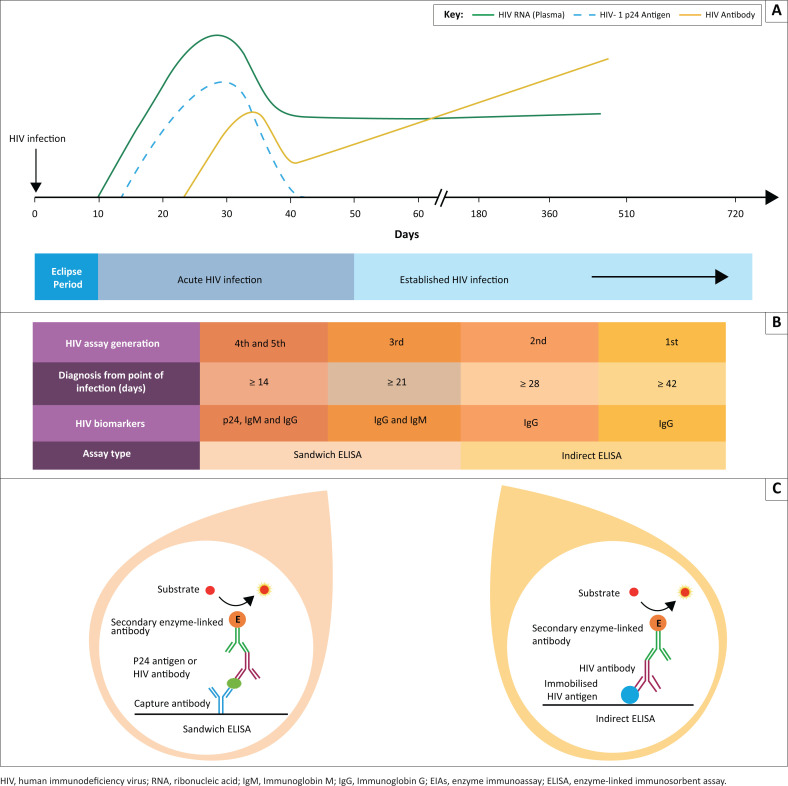
Laboratory diagnosis of HIV infection in adults. (a) HIV diagnosis is made by detecting HIV biomarkers: HIV nucleic acid, RNA, HIV-1/2 p24 and HIV antibodies (Immunoglobin M and Immunoglobin G). (b) Technological evolution in HIV assay design has enabled early diagnosis from the point of infection. (c) Enzyme immunoassay used in HIV testing utilise enzyme-linked immunosorbent assay design. First- and second-generation HIV EIAs employ indirect enzyme-linked immunosorbent assay format in contrast to the sandwich enzyme-linked immunosorbent assay applied in the third-, fourth- and fifth-generation HIV assays.

The definitions of acute, early, chronic and late infection consider variable factors. These include evolution of HIV viraemia, HIV antigenemia, HIV antibody responses, recognition of a stable set point viral load and the onset of clinical immunodeficiency.^5,10,11,12^ HIV antibodies (IgG and IgM) and the p24 biomarker are used in the diagnosis of HIV infection, and these biomarker profiles change from the point of infection (Figure 1).

The eclipse phase of HIV infection is characterised by the inability of laboratory tests to detect HIV. Therefore, an infected person will test negative during the eclipse phase.^11^ HIV EIA tests detect antibodies produced against HIV and/or p24 HIV antigen. The EIA tests are formatted as enzyme-linked immunosorbent assays (ELISAs). Enzyme-linked immunosorbent assays can detect HIV antibodies and/or p24 proteins. HIV ELISAs can be categorised into two formats: the indirect ELISA format is used in first- and second-generation HIV assays, and sandwich ELISA format is used in the third-fifth generation HIV assays (Figure 1). Enzyme-linked immunosorbent assays utilise capture proteins (HIV viral antigen) or capture antibodies, which entrap HIV antibody or HIV antigen, respectively. Secondary antibodies conjugated to enzymes detect the captured p24 antigens or HIV antibodies by an enzyme-catalysed reaction of an added substrate. A measurable quantifiable signal is produced that indicates the presence or absence of HIV biomarkers.^13,14^

Enzyme immunoassay testing has undergone a progressive evolution over the last 25 years (Figure 1). First- and second-generation assays detect IgG against HIV-1 infection at approximately 6–10 and 4–6 weeks, respectively.^15^ The third-generation assay also detects IgM and IgG against HIV-1, and a diagnosis can be made from 3 weeks post-infection.^16^ Around 2010, the newer fourth-generation assay replaced third-generation tests by combining the detection of early p24 and HIV antibodies (IgM and IgG). This resulted in HIV detection as early as 2 weeks post-infection.^17^ The HIV p24 antigen can also be used to detect HIV infection in late disease when the HIV antibody response wanes accompanied by HIV viraemia. In contrast to the fourth-generation assay, the fifth-generation assay quantifies HIV antibodies and p24 separately.^18^ HIV-1 and HIV-2 can be detected by the third-, fourth- and fifth-generation assays. In summary, the evolution of HIV assays has narrowed the window period for early HIV diagnosis.

## Measuring the performance of an HIV test

HIV testing is evaluated by the EIA diagnostic performance for HIV infection and non-infection. A screening test for HIV needs to be highly sensitive to detect all true-positive cases of HIV infection and therefore accurately reflect the number of positive HIV test results in patients infected by HIV (true positives). In contrast, the specificity of the test defines the number of negative test results in patients who have not been infected by HIV (true negatives). In all analytical testing systems, there is a trade-off between sensitivity and specificity of a test result.^19,20^ The sensitivity and specificity of the test thus provide an overall accuracy for the screening and diagnosis of HIV. The fourth-generation laboratory-based assays demonstrate sensitivity of ≥ 99.9% and specificity ≥ 99.5%, whilst the fifth-generation assay has shown 100% sensitivity and 99.5%, specificity.^20,21^ Minor variations in diagnostic performance of laboratory-based fourth- and fifth-generation assays occur between manufacturer kits.

In high prevalence settings, HIV diagnostic tests produce high positive predictive values for HIV infection. In SA, all screening tests undergo confirmation testing and subsequent work-up for patients suspected of HIV infection and are guided by the National HIV Testing Services policy.^22^

In clinical practice, one may encounter false-positive and/or false-negative HIV test results. Whilst these were more common with early generation tests, false results may still be reported using fourth and fifth generation assays.^23,24^ Errors can be caused at various steps in the laboratory testing process (Table 1).^24,25,26^ These include steps before the patient specimen arrives at the laboratory (pre-analytical), for example, sampling errors, or during analysis of the patient specimen (analytical), for example, test kit variability or less commonly, after the specimen has been tested (post-analytical). Moreover, timing of testing and insufficient antibody titres may also cause false results.

**TABLE 1:** Causes of false serology test results.

False negative	False positive
**1. Pre-analytical error** Mislabelled sample tubes Expired samples Insufficient sample Incorrect sample tube	**1. Pre-analytical error** Lipaemic, haemolysed or icteric specimens Mislabelled samples
**2. Analytical error** Variability between test kits to detect HIV subtypes Use of expired reagents or test devices Sub-optimal transport and storage of assays causing assay degradation Manufacturing error because of lapse in quality management system	**2. Analytical error** Cross-reacting antibodies with ELISAs Renal failure Pregnancy Myeloma Recent vaccination, for example, influenza Blood transfusion Transplantation Other infections: viral, bacterial or parasitic Autoimmune disease Antibody interference with ELISAs Heterophile antibodies Human anti-mouse antibodies Cross-reactive antigens Contaminating proteins in the sample Manufacturing defects because of lapse in quality management system Contamination with HIV biomarkers Pipetting errors On-instrument HIV contamination
**3. Biological error** Ongoing seroconversion Sampling during the window period Divergent HIV strains	**3. Post analytical-error** Over-interpretation of weakly reactive test lines on visually read assays
**4. Insufficient antibody titres** Seroreversion Advanced disease (CD4 < 200 cell/mm^3^) Elite controller Early exposure to HIV antiretroviral drugs (e.g. pre- and post-exposure prophylaxis)	-

*Source:* Please see the full reference list of the article Liu P, Jackson P, Shaw N, Heysell S. Spectrum of false positivity for the fourth generation human immunodeficiency virus diagnostic tests. AIDS Res Ther. 2016;13:1. https://doi.org/10.1186/s12981-015-0086-3, for more information.

HIV, human immunodeficiency virus; ELISA, enzyme-linked immunosorbent assay; CD4, cluster of differentiation 4.

## Rapid HIV tests

Rapid tests (RTs) are a simplified version of an HIV ELISA and can detect p24 antigen and/or HIV antibodies. The South African National testing services policy, which reflects the World Health Organization (WHO) guidelines, recommend that adults are screened for HIV at the point of care using two RTs by a serial testing algorithm.^27^ This means that if the first test is reactive, it is followed by another RT from a different manufacturer to confirm the first screen reactive result. Alternatively, if the screening test yields a non-reactive result, it should be reported as negative. Discordant test results, that is, where the first result is reactive and the second result is non-reactive, should prompt a repeat RT following the serial national HIV testing algorithm. A laboratory HIV test is indicated in the case of persistently discordant results.

The advantage of HIV RTs is that it may be performed by non-laboratory staff and is usually completed in less than 30 min. Minimal equipment is required, and the test may be performed on finger-prick blood or oral fluid. This enables a result to be obtained at the time of consultation in various settings.^28,29,30^

A disadvantage of RTs is decreased sensitivity compared to laboratory tests as blood from a finger-prick capillary sample has a lower concentration of antibodies and p24 antigen than a serum or plasma sample. Consequently, the detection of HIV is likely to lag by a few days in comparison to a laboratory test. Moreover, the HIV RTs are not as sensitive in comparison with higher test diagnostic accuracy of laboratory-based tests for early HIV infection.^31,32^

In SA, the state sector utilises the third-generation RTs to screen for HIV.^33^ This can cause a false-negative diagnosis, delay initiation of antiretrovirals, increase HIV transmission and delay contact tracing.^34^ Of note, the fourth-generation RT does not perform superiorly to the third-generation rapid assay in detecting early HIV infection.^35^ Therefore, an improved fourth-generation RT, for key high incidence populations such as men who have sex with men (MSM) and those on pre-exposure prophylaxis (PrEP), may provide an advantage by identifying early HIV infection. In 2015, Alere released a re-formulated fourth-generation RT kit Alere™ HIV Combo. In the Vaginal and Oral Interventions to Control the Epidemic (VOICE) study, 28% of infections missed by current third-generation RTs would have been identified with the use of the Alere™ HIV Combo.^36^ Further field research examining the feasibility and utility of this RT as part of the testing algorithm in SA is warranted.

## HIV self-testing

HIV self-testing (HIVST) is a procedure whereby a person collects their own sample, performs the test and interprets the result on their own.^37^ Samples that may be used include finger-prick blood samples and oral fluid. A self-test is intended to be used by an untrained person and should be easy to use with clear instructions. Some devices are based on second-generation HIV assays and therefore less likely to detect early HIV infection. The OraQuick HIVST, currently used in SA, is based on a third-generation test. The HIV Self-Testing Africa (STAR) Initiative is a 5-year project working with national health authorities in six participating Southern African countries to scale up HIVST. Partners include the Wits Reproductive Health and HIV Institute (Wits RHI) and Society for Family Health (SFH). Only HIVSTs with a WHO pre-qualification are procured in SA.^38^ A list of approved HIVSTs and those in the pipeline can be viewed at https://unitaid.org/assets/HIVST-landscape-report.pdf.^39^ Distribution channels comprise health clinics, workplace programmes and over-the-counter sales.

Target populations include sex workers, MSM, transgender people, people who inject drugs and mobile populations. Those on PrEP and in HIV-related clinical trials should not use HIVSTs because of potential false non-reactive outcomes. A non-reactive self-screening result should prompt a retest after 6 weeks with linkage to HIV prevention services, whilst a positive result requires further testing and management by a trained healthcare provider. Linkage strategies after HIV self-screening include a telephone hotline for referral and information contained in a care card.^38,40^ Community-based follow-up provides additional post-screen counselling with referral to confirmatory testing services.

## Conclusion

The early diagnosis of HIV in adults has been substantially improved with advancements in EIA HIV-testing technology. Enzyme immunoassay, however, still suffer from false-positive and -negative results and require careful interpretation together with clinical and laboratory inputs to correctly inform the diagnosis of HIV infection.
